# Identifying Risk Factors for *Stephanofilaria*-Caused Ulcerative Dermal Lesions, in Black and White Rhinoceros' Meta-Population in Kenya

**DOI:** 10.1155/2024/2655970

**Published:** 2024-01-10

**Authors:** Edward King'ori, Grace Waiguchu, Mukami Ruoro, Kenneth Muriithi, Cecilia Mumbi, Martin Omondi, Duncan Aminga, Shaleen Angwenyi, Domnic Mijele, Patrick I. Chiyo

**Affiliations:** ^1^Veterinary Department, Kenya Wildlife Service, P.O. Box 40241-00100, Nairobi, Kenya; ^2^Research Division, Wildlife Research and Training Institute, P.O. Box 842-20117, Naivasha, Kenya; ^3^Wildlife Security Directorate, Kenya Wildlife Service, P.O. Box 40241-00100, Nairobi, Kenya; ^4^Natural State Research Centre, Private Bag, Isiolo, Kenya; ^5^Global Health Program, Smithsonian Conservation Biology Institute, Washington, DC, USA; ^6^Wildlife Genetics and Forensics Laboratory, Kenya Wildlife Service, P.O. Box 40241-00100, Nairobi, Kenya

## Abstract

Ulcerative skin lesions caused by *Stephanofilaria dinniki* are common in populations of the critically endangered eastern black rhinoceros (*Diceros bicornis michaeli*). Although considered benign, they have been associated with loss of body condition and mortality in rhinoceros. Stephanofilarial lesions in rhinoceros may also lead to delayed puberty, reduced milk yields, and prolonged intercalving periods as observed with a similar disease in cattle. In this study the prevalence and predilection sites of stephanofilarial wounds were examined and the influence of age, sex, normalized difference vegetation index (NDVI), rainfall, temperature, rhinoceros' species, and population density on the prevalence of stephanofilarial skin lesions was evaluated in 10 rhinoceros' populations in Kenya. The results revealed that the prevalence of stephanofilarial skin lesions was 0% in the lowland sanctuaries such as Ngulia Rhino Sanctuary, Tsavo West Intensive Protection Zone, and Tsavo East National Park to ≥90% in the highland rhinoceros' sanctuaries including Solio Wildlife Sanctuary, Ol Pejeta Wildlife Conservancy, Lewa-Borana Wildlife Conservancies, and Nairobi National Park. Generalized linear models revealed that monthly minimum temperature and NDVI negatively influenced the probability of stephanofilarial skin lesions. However, spatial heterogeneity in NDVI, and rhino density were positively related to the probability of rhinoceros' infection with *Stephanofilaria*. Black rhinoceros had a higher average prevalence of stephanofilarial wounds (50.9%, *n* = 228) than white rhinoceros' (3.6%, *n* = 110). Temperature and heterogeneity in NDVI can directly influence the presence of the filaria vector *Rhinomusca dutoiti* as corroborated by the previous studies on their distribution. Moreover, the inverse relationship between NDVI and the prevalence of stephanofilarial skin lesions suggests that nutrition negatively impacts rhinoceros' immunity. Comprehensive studies on the vectors of *Stephanofilaria* and their bionomics may illuminate the epidemiological patterns of stephanofilariosis in rhinoceros.

## 1. Introduction

Ulcerative filarial skin lesions caused by a host specific nematode, *Stephanofilaria dinniki* [1, 2] is a common disease in the critically endangered eastern black rhinoceros (*Diceros bicornis michaeli*) but has recently been documented in the endangered southern white rhinoceros (*Ceratotherium simum simum*) [3]. These ulcerative lesions present as crater-like, open hemorrhagic wounds that often vary in the surface area of skin affected. Although, these stephanofilarial infections have been considered benign in black rhinoceros, they may impact host populations by exacerbating the effects of seasonal and limited forage availability on the body condition, fecundity, and survival of individuals with implications for species recovery from extinction [4–6]. A similar disease in cattle caused by a related nematode *Stephanofilaria assamensis*, has been implicated in delayed puberty, reduced lactation yields, and prolonged intercalving period in the affected cattle [7]. Recent studies have shown that these wounds are associated with a loss in body condition, anemia, elevated stress, and mortality in black rhinoceros [3, 8] and may be chronic [9]. These hemorrhagic skin lesions can cause black rhinoceros' mortality through systemic secondary bacterial infection. Flaring up of wounds can also be induced by translocation-associated stress particularly in artificially managed rhinoceros' metapopulations [10].

*Stephanofilaria* species in livestock are transmitted by different hematophagous flies including the buffalo fly *Hematobia irritans exigua* [11], *Haematobia atripalpis*, *Haematobia (Lyperosia) titillans*, and *Haematobia (Lyperosia) irritans* [12]. Although, no studies have been undertaken to identify the vectors of *S. dinniki*, several fly species associated with rhinoceros in East and Southern Africa are well-characterized and described [13, 14]. *Rhinomusca dutoiti* and *Rhinomusca brucei* fly species have been suspected to be vectors for *S. dinniki* because of their specific association with hemorrhagic filarial lesions in East and Southern Africa black rhinoceros [13–16]. Another potential vector of *Stephanofilaria* is a buffalo fly of the genus *Haematobia (Lyperosia*) [13] known to swarm rhinoceros in Eastern Africa and are known vectors of other species of *Stephanofilaria* in livestock [11, 12, 17].

The prevalence of filarial lesions is known to vary across rhinoceros' populations [8, 9, 16]. Despite this variation, there are no systematic studies of potential drivers of this observed variation. Like most vector borne diseases, the factors that influence the presence and abundance of the vector may be key drivers of disease prevalence patterns [18]. Temperature, humidity, and rainfall are known to play an important role in the distribution and abundance of the vectors, survival of the pathogens, and their transmission efficiency [19]. Vegetation structure and productivity measured using remotely sensed normalized difference vegetation index (NDVI) is another variable known to influence the abundance of disease vectors [19, 20] and the nutritional status of the host with implications for disease epidemiological patterns [21]. Other factors that may influence disease prevalence patterns include individual traits such as age, sex, behavior, and their nutritional status as these may influence exposure to pathogens and susceptibility to infection [21–24]. Host population density is another factor that is known to affect disease prevalence as it increases the pool of maintenance hosts and or increases vector-host encounter rates [25–27]. Moreover, studies on *Stephanofilaria* infections in cattle have demonstrated consistent predilection sites for skin lesions associated with the different species of *Stephanofilaria* [28], but only a single study has examined predilection sites of *S. dinniki* in black rhinoceros in southern Africa [16].

Despite knowledge of potential vectors for *Stephanofilaria*, the influence vegetation structure and productivity on the survival and abundance vectors of *Stephanofilaria*, and the nutritional status of the host, and how these inturn influence patterns of rhinoceros stephanofilaria infections are not known. Similarly, the role of animal traits like sex and age, also considered important determinants of infection with stephanofilarial skin lesions in livestock, are not documented in wildlife species specifically rhinoceros. In addition, there is a lack of baseline data on prevalence and predilection sites of stephanofilarial skin lesions in East African rhinoceros. In this study, we obtained baseline data on the prevalence and predilection sites of filarial wounds and assessed the influence of age, sex, NDVI, rainfall, temperature, species, and host population density on the prevalence of stephanofilarial skin lesions in 10 rhinoceros' populations in Kenya. Additionally, we examined patterns of clinical interventions on black and white rhinos due to stephanofilarial skin lesions as these would indicate the severity or debilitating effects of disease within and between populations, sexes, and species.

## 2. Methods

### 2.1. Ethics Statement

This study was approved by the Ethics Committee of the Kenya Wildlife Service (KWS/BRM/5001), the institution mandated to protect and conserve Wildlife in Kenya. All data used were collected during immobilization for management interventions such as ear notching, translocation, clinical management of injuries (due to snares and intraspecific fights), and clinical infections. Immobilization and translocation are undertaken by experienced veterinarians from the Kenya Wildlife Service (KWS) guided by the KWS protocol for rhinoceros' immobilization and translocation, guidelines on Wildlife Veterinary Practice 2018 and the Veterinary Surgeons and Veterinary Para-professionals Act Cap 366 of the Laws of Kenya that regulates veterinary practice in Kenya.

### 2.2. Study Area

**Figure 1:**
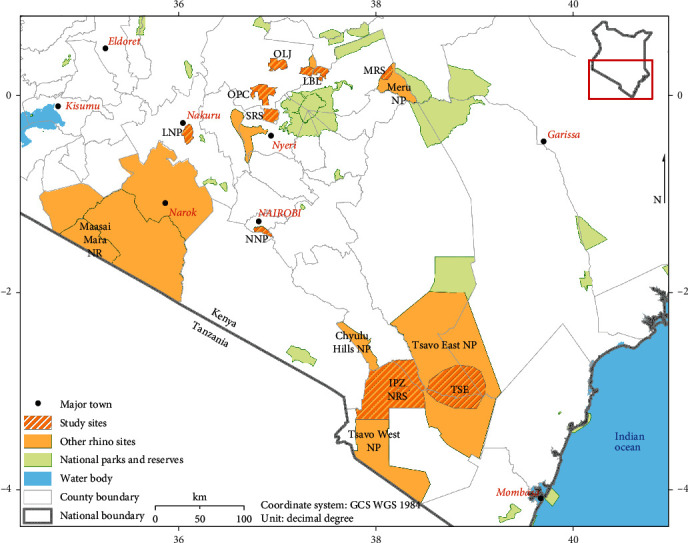
Map of Kenya showing all rhinoceros subpopulations and those sampled during this study.

Rhinoceros were sampled from 10 populations in Kenya (Figure 1) including the following: Lake Nakuru National Park (LNP), Nairobi National Park (NNP), Ngulia Rhino Sanctuary (NRS), Ol Pejeta Conservancy (OPC), Ol Jogi Conservancy (OLJ), Lewa–Borana Conservancy Landscape (LBL), Meru Rhino Sanctuary (MRS), Solio Rhino Sanctuary (SRS), Tsavo West Intensive Protection Zone (IPZ), and Tsavo East National Park (TSE).

LNP located at 00°50 S–01°00′ S and 36°20′E–36°25′ E, has a perimeter fence around the rhino sanctuary and covers 188 km^2^ with an altitude ranging from 1,200 to 1,750 m. It receives an average annual rainfall of 850 mm and has vegetation composed of a mixture of open grassland *Acacia*, *Tarchonanthus* bushland, deciduous and *Euphorbia* forests, and riverine bushland. NNP located at 36°23° E–36°28E and 2°18′ S–2°20′ S is partially fenced with unfenced 20 km stretch along the southern boundary to allow wild ungulate migration. NNP covers about 117 km^2^ with an altitude ranging from 1,508to 1,790 m and receives an average annual rainfall of 800 mm. Its vegetation comprises deciduous forest, riverine thorn forests, shrubs, and grasslands. NRS is located at 38°06′E–38°10′E and 3°01′S–3°06′S inside Tsavo West National Park and covers an area of about 90 km^2^ enclosed by a perimeter fence. NRS ranges in altitude from 600 to 1,800 m and receives an average rainfall of 600 mm per annum. The vegetation of NRS is composed of mixed species bushland thickets, grasslands, shrubs, low tree, and herbs. OPC covers 93 km^2^ enclosed in a perimeter fence. OPC is situated in central Kenya between 36°40′E–37°°00′E and 0°02′S–0°07′ N. OPC has an undulating terrain ranging between 1,770 and 1,820 m in altitude. OPC receives on average 850 mm of rainfall per annum. OPC has a mosaic vegetation consisting of grassland, *Acacia* woodland, *Euclea* shrub, and riverine woodland. OLJ is a completely fenced 235 km^2^ reserve on private land located in central Kenya between 37°00′E–37°05′E and 0°15′N–0020′N. The terrain undulates between 1,800 and 1,920 m altitude. Rainfall averages 460 mm per year. The vegetation is a mosaic of grassland, Acacia woodland and shrubs. Lewa Wildlife Conservancy and Borana Wildlife Conservancy, two rhino conservancies which recently created a joint perimeter fence for rhino protection in an area of 376.36 km^2^ and form the LBL. LBL lies between 00°10′N–0015′N, and 37° 15′E–37°20′E with an altitude ranging between 1,750 and 1,950 m altitude and with an average rainfall of 550 mm per year. The vegetation of LBL is dominated by *Stipa dregeana* forest, *Acacia-Commiphora* woodlands and open grasslands. MRS is a fenced portion in the Western part of Meru National Park covering 38.8 km^2^ park and located between 36°40′ E–37°00′ E and 0°02′ S–0°07′ N. Meru National Park covers an area of 870 km^2^ and receives rainfall ranging from 635 to 762 mm in the west of the park to 305–356 mm in the east. SRS covers 76.9 km^2^ in the Laikipia–Samburu ecosystem (0°16′S–0°27′ S and 36°53′E–37°00′ E), which is characterized by savannah grassland dominated by *Euclea divinorum*, Acacia and Euphorbia woodlands. Annual rainfall averages 300–700 mm and has an altitude of 1,932 m, located at the base of the Aberdares Mountains. IPZ located in Tsavo West National Park at 37°45′ E–38°45′ E and 2°40′ S–4°02′ S and covers an area of 3,000 km^2^. It is considered the most viable habitat in Kenya for rhinoceros population recovery. TSE lies along 38°10′ E–39°25 E and 1°50′S–3°30′S and covers an area of 13,747 km^2^. The rhinoceroses occur South of the Galana River and North of the Voi River, in an area of about 3,300 km^2^. The altitude in the southern part rises from 150 m to the East and to 1,200 m in the Yatta Plateau in the west. Annual mean rainfall in the park varies according to altitude with the eastern part receiving about 250 mm while the western part receives about 450 mm. The park is sparsely vegetated with extensive grassland and Bushland savannahs comprising semi-arid Acacia–Commiphora woodlands with *Premna*, *Bauhinia*, and *Sericocomorpsis* scrub interspersed with *Delonix elata* and *Melia volkensii* trees.

### 2.3. Prevalence Patterns of Filarial Skin Lesions in Rhinoceros

The Kenyan rhinoceros population consists of 17 spatially separated subpopulations managed collectively as one metapopulation through occasional movement of animals across subpopulations and creation of new subpopulations from existing constituent populations. The 17 rhinoceros' subpopulations are mostly in fenced sanctuaries spread across the country (Figure 1). According to the 2021 National Wildlife Census, Kenya had 897 black rhinos and 842 white rhinos. We sampled 228/897 black rhinoceros and 110/842 white rhinoceros or 25.4% and 13.1% of the species populations, respectively. The proportions of animals sampled in each population are indicated in Table 1.

The presence of filarial skin lesions in rhinoceros was recorded during routine management activities such as ear notching, rhino censusing, individual rhinoceros monitoring and clinical intervention on animals with ailments for treatment or de-snaring of individual animals. The bulk of the data was collected during ear notching exercises (a management practice used to uniquely identify individual animals and is used in monitoring of rhinoceros subpopulations), and sighting of individuals in populations as part of rhinoceros monitoring programs. This approach was used to monitor infection patterns because rhinoceros are elusive animals and random sampling would be an impossible approach. Moreover, each of these methods depend on opportunistic sighting of individuals and any resulting bias will cut across all populations studied and should not affect our analyses of variables influencing disease prevalence patterns in these populations. In 2021, four rhinoceros' ear notching exercises were conducted: twenty-two (22) black rhinoceros and six (6) white rhinoceros were ear-notched in NNP, 14 black rhinoceros and 6 white rhinoceros in LNP, seven (7) black rhinoceros and 24 white rhinoceros in MRS, and seventeen (17) black rhinoceros in NRS. Three rhinoceros' ear notching exercises were conducted in 2022: thirty-one (31) rhinos were notched in OLJ, twenty (20) black rhinos, and eleven (11) white rhinos. Thirty-one (31) black rhinos were notched at IPZ, twenty-four (24) black rhinos were notched in TSE. In 2022, observations on stephanofilarial wound data were also recorded during routine monitoring in three populations: seventeen (17) black rhinos and sixteen (16) white rhinos were observed in SRS and eleven (11) black rhinos were observed in NNP. Two rhinoceros' ear notching exercises were undertaken in 2023; twenty-four (24) black rhinos and twenty-one (21) white rhinos were notched in OPC while twenty-three (23) black and twenty-one (21) white rhinos were notched at LBL. Summaries are presented in Table 1.

For each individual rhinoceros handled, the following information was recorded: sex, ID, age, predilection site of filarial skin lesions and species (black rhinoceros or white rhinoceros) and the presence or absence of filarial skin lesions.

**Figure 2:**
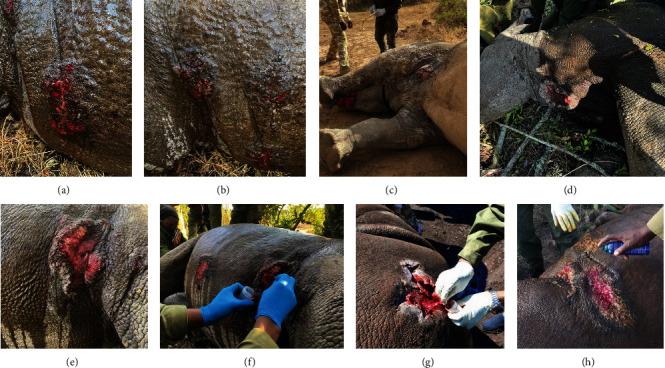
Photos of stephanofilarial wounds in rhinos showing early stage (a) filarial wounds and late stage or healing wounds (h) and intermediate stages (b–g). Notice the raised and crusting wounds, mottled appearance, and crater-like wounds (g). f and g show wound scrapings being taken for worm isolation and detection.

Stephanofilarial lesions are easily distinguishable from other skin lesions as described elsewhere [2, 3, 16, 29]. Generally, they are crater-like (raised at edges), open hemorrhagic wounds that exhibit exfoliative crust formation with a rough mottled appearance (Figure 2). Scrapings from active wounds were scanned in a dissecting microscope and worms were isolated and examined. Morphological and genetic analyses described elsewhere (manuscript in preparation) confirmed the worms observed are *S. dinniki*.

The rhinoceros age was determined from estimated date of birth based on the last date a pregnant rhinoceros was observed and the first date the mother and calf are observed in routinely monitored populations. For less monitored populations, animals were assigned into age classes using field age assignment criteria for black [30] and white [31] rhinoceros. Generally, animals were classified into three age categories: AD = Adults (more than 7 years of age), SA = Sub-Adults (between 3.5and 6.9 years of age), CA = Calves (less than 3.5 years of age) based on relative body size, shape, and relative horn length [32].

Species identity was determined using relative body shape, behavior, and shape of lips [33, 34]. White rhinoceros have a flat back and bump on the lower body while black rhinoceros possess a deep arched back. White rhinoceros have flat and broad lips while black rhinoceros have a pointed prehensile lip (with the shape of a hook) adapted to feed on grass and to pluck leaves from shrubs and trees, respectively. The white rhinoceros has smaller, round ears while the white rhinoceros has long trumpet-shaped ears. Rhinoceros were individually identified using unique ear notch sequences whenever available. When not ear notched, rhinoceros were identified using sex, age, horn shape and length, natural ear notches and deformities, body scars and corrugations, tail shape and size, nose and eye wrinkles, and mother and calf association [35–37]. However, when skin lesions were present, we recorded the location or part of the body affected.

### 2.4. NDVI, Precipitation, and Temperature

#### 2.4.1. Precipitation Metadata

Precipitation data were obtained from the Copernicus Climate Change service [38], climate data store, accessed through Google Earth Engine (GEE) [39]. Rainfall estimates are calculated in a model grid box over a spatial resolution of 11,132 m by accumulated liquid and/or frozen water that falls on the earth's surface. A sum of the daily precipitation was extracted for the central location on each study site. Coordinates for the most central location were acquired by running the tool “polygon to point” on ArcMap 10.4 (ESRI).

#### 2.4.2. Temperature Metadata

The temperature data were also derived from the Copernicus Climate Change service [38], climate data store, accessed through GEE. It is calculated by interpolating between the lowest model level and the Earth's surface, taking account of the atmospheric conditions to get the temperature of air at 2 m above the surface of land. The daily data were extracted for the most central location within each study site, at a spatial resolution of 11,132 m^2^.

#### 2.4.3. NDVI Data

NDVI data were obtained from the Modis Terra Vegetation Product 16-day global dataset with a spatial resolution of 250 m [40] on GEE [39]. It is derived from the existing National Oceanic and Atmospheric Administration-Advanced Very High-Resolution Radiometer (NOAA-AVHRR) and computed from the atmospherically corrected bidirectional surface reflectance that have been masked for water, clouds, heavy aerosols, and cloud shadows. The downloaded product was processed in ArcGIS 10.4 (ESRI) using the extract multivalues from raster tool to extract the average monthly NDVI values for 30 random points in each study site.

## 3. Statistical Analyses

To test whether filarial wound predilection sites were homogeneously distributed across body sites in both white and black rhinoceros' skin, a *χ*^2^ Test was used. The variation in filarial wound predilection sites between rhinoceros' species and across locations was tested using the exact two-sample Kolmogorov–Smirnov Test. Variation in monthly environmental variables (temperature, rainfall, and NDVI) between sanctuaries, and sampling years (2021–2023) was examined using two-way ANOVA. Data years were defined as starting in June of a one calendar year and ending in May of another calendar year.

Prevalence patterns of stephanofilarial wounds for each rhinoceros species and sanctuary were determined as the proportion (expressed as a percentage) of all observed rhinoceros that had wounds. For each prevalence value, the 95% confidence interval for a binomial probability was determined using the formula: confidence interval = *p* ± z^*∗*^(√*p*(1−*p*)/*n*), where: *p* is the proportion of rhinoceros with stephanofilarial wounds, *z* is the *z*-value for a 95% confidence interval (1.96), and *n* is the sample size or total number of rhinoceros examined.

We used a generalized linear model (GLM) with a logit link and a binomial error to test the influence of monthly NDVI, spatial heterogeneity in monthly NDVI and ambient monthly temperature metrics (minimum, maximum, and mean) and monthly rainfall at the location of rhinoceros sampling as independent variables on the probability of presence of stephanofilarial wounds (as a dependent variable) on rhinoceros in Kenya. We also tested the influence of individual rhinoceros' specific traits such as sex, age class (Adult, Sub-adult, and calf), species (white or black rhinoceros) on the probability of infection. Lastly, we also tested for density dependence on infection probability by using combined black and white rhino density as an independent covariate. To evaluate the predictive value of the independent variables, GLM was performed on a permutation of all possible covariate combinations and the best model was selected based on AICc using MuMIn R package [41]. To avoid the effects of multicollinearity, we used models with one of the collinear independent variables included minimum, mean, or maximum temperature and mean NDVI or total rainfall. The best model was selected from the subsets with the smallest AICc. For the best model, the area under the curve (AUC) of the ROC (receiver operating characteristic) curve was calculated as it provides an overall measure of model fit or a measure of the separation between classes in a binary classifier. As a rule of thumb a model with an AUC between 0.8 and –0.9 is considered excellent and a model with an AUC above 0.9 is considered outstanding [42]. We also determined several pseudo *R*^2^ for the fitted model to assess fit. For example Mcfadden *R*^2^ of 0.2–0.4 is considered as an excellent fit [43].

All the statistical analyses mentioned above were performed using the R software (R version 4.3.1) for statistical computing [44].

## 4. Results

### 4.1. Predilection Sites for Filarial Wounds in Kenyan Rhinoceros

**Figure 3:**
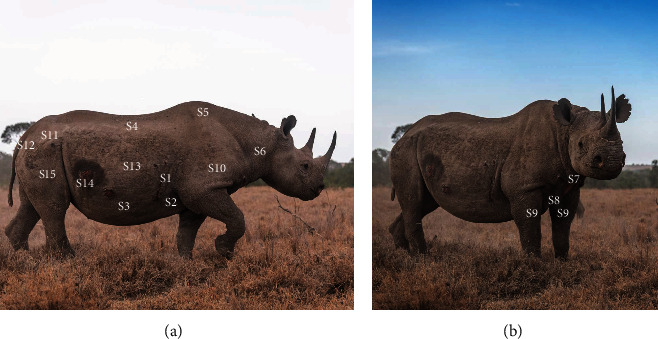
(a, b) The distribution of filarial wound predilection sites on black rhinoceros (active wounds can be seen next to S14 and S1).

The distribution of filarial wounds was not random in both black (*χ*^2^_14_ = 454, *P*  < 0.0001) and white (*χ*^2^_14_ = 35, *P*=0.001) rhinoceros. Most filarial wound sites were located around the chest with 55% of individuals having lateral–posterior chest wounds and nearly 50% having lateral–anterior lower and upper chest wounds (Figure 3 and Table 2) in black rhinoceros. In white rhinoceros, filarial wounds were common on the hind legs particularly the thighs.

Exact two-sample Kolmogorov–Smirnov test revealed a statistically significant difference in the distribution of filarial wound predilection sites between white and black rhinoceros (*D* = 0.467, *P*=0.042). However, there was no statistically significant variation in the distribution of filarial wound predilection sites across rhinoceros' sanctuaries. Pairwise comparisons among sanctuaries with many positive samples revealed that the distribution of predilection sites between rhinos in OPC and NNP (*D* = 0.3, *P*=0.2), LBL and NNP (*D* = 0.2, *P*=0.8), and LBL and OPC (*D* = 0.3, *P*=0.2) were not statistically different from each other.

### 4.2. Spatial and Species Variation in the Prevalence of Filarial Wounds in Kenyan Rhinoceros

Prevalence of filarial wounds was generally higher in black rhinoceros (50.88%) than in white rhinoceros (3.64%). In black rhinoceros, the prevalence of filarial wounds in the 10 rhinoceros sanctuaries examined showed extreme variability in filarial wound infection patterns (Table 3). Some sites, particularly those in the Tsavo conservation area, namely NRS, IPZ, and TSE; and LNP rhinoceros sampled had no filarial wounds (Table 3). On the other hand, SRS, LBL, NNP, and OPC had prevalence of filarial wounds in 90%–100% of rhinoceros. At intermediate prevalence were the rhinoceros' populations from MRS and OLJ (Table 3). In white rhinoceros, the prevalence of filarial wounds was generally low and occurred only in SRS and MRS.

### 4.3. The Influence of NDVI, Rainfall, Temperature, Rhinoceros' Species, Sex, and Age on the Prevalence of Filarial Wounds in Kenya's Rhinoceros

Mean rainfall was highly variable across rhinoceros' sanctuaries being lowest in TSE and highest in SRS (Table 4). Mean temperature followed an opposite trend, being lowest at SRS and highest at TSE (Table 4). Minimum and maximum temperatures follow a similar trend to mean temperature. NDVI was highest in LNP and lowest in TSE. Vegetation heterogeneity as measured using NDVI was highest in NNP followed by SRS and MRS and lowest at OLJ, NRS, and LBL (Table 4). Patterns of environmental variation across rhinoceros' populations were similar to a 7-year average (Table S1).

A two-way ANOVA to examine the effect of sanctuary, and sampling year on monthly minimum temperature revealed a significant influence of sanctuary (F_(9)_ = 469.38, *P*  < 0.0001), but not year (F_(2)_ = 2.27, *P*=0.105) on mean monthly minimum temperature. A similar pattern of variation was observed for mean maximum temperature between sanctuaries (F_(9)_ = 209.68, *P*  < 0.0001), as well as between years (F_(2)_ = 5.26, *P*  < 0.0056). Tukey HSD pairwise comparisons of maximum and minimum temperature among sanctuaries revealed that some locations were similar in temperature. There were three groups of sanctuaries which had similar minimum temperatures. The first group consisted of LBL, LNP, and OLJ and the second group had SRS and OPC, while the third group included TSE and MRS. NRS and IPZ were high and NNP had low but distinct temperatures. Pairwise comparisons in relation to maximum temperature also revealed two distinct groups based on similarity: NNP, LNP, OPC, and OLJ as one group and TSE and MRS as another. Mean rainfall was also variable between sanctuaries (F_(9)_ = 17.06, *P* < 0.0001) and sampling year (F_(2)_ = 9.75, *P*  < 0.0001). NDVI followed a similar pattern of variation between sanctuaries (F_(9)_ = 18.39, *P*  < 0.0001) and years (F_(2)_ = 14.12, *P*  < 0.0001). However, mean spatial heterogeneity in NDVI varied between sanctuaries (F_(9)_ = 11.84, *P* < 0.0001), but not years (F_(2)_ = 2.12, *P*=0.121).

The best model based on AICc Criteria (Table 5) indicated that the factors with the greatest influence on the probability of rhinoceros infection with *Stephanofilaria* are minimum monthly temperature, NDVI, heterogeneity in NDVI, rhinoceros' density, and rhinoceros' species. This model had an AUC of 0.94 and the *R*^2^ values were as follows: Nagelkerke's *R*^2^ (Cragg–Uhler) was 0.66, the coefficient of discrimination D or Tjur's *R*^2^ was 0.59, and McFadden's pseudo-*R*^2^ was 0.51 all suggesting excellent model fit.

**Figure 4:**
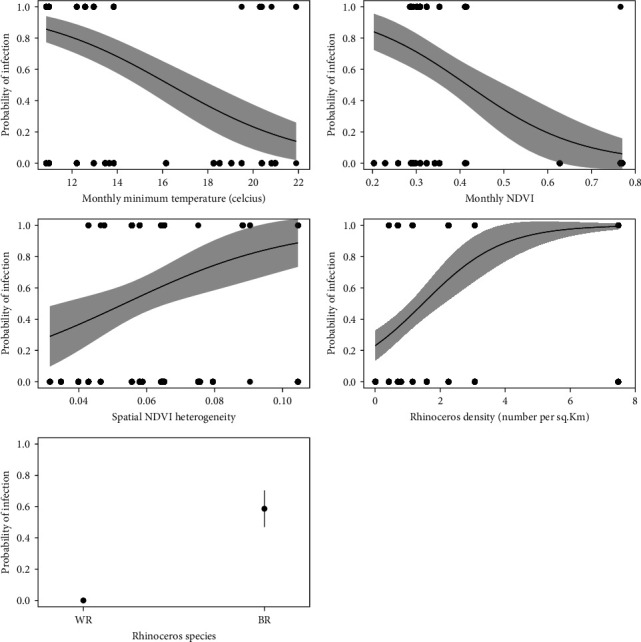
The influence of NDVI, spatial heterogeneity in NDVI (SD (NDVI)), minimum temperature, rainfall, rhinoceros' density, and rhinoceros species identity (BR is *Diceros bicornis michaeli* and WR is *Ceratotherium simum simum*) on the probability of infection with *stephanofilaria dinniki*.

The mean minimum temperature at the month of sampling was negatively related to the probability of *Stephanofilaria* lesion presence with low minimum temperature being associated with a higher probability of *Stephanofilaria* lesion presence than higher minimum temperature (Figure 4). Similarly, low NDVI at the month of sampling was associated with a higher probability of stephanofilarial infection in rhinoceros than high NDVI. However, spatial heterogeneity in NDVI was positively related to higher probability of *S. dinniki* infection in rhinoceros (Figure 4). Stephanofilarial skin lesions were density dependent, with a higher probability of infection occurring in high-density populations than in low-density populations. Black rhinoceros were more infected with *Stephanofilaria* than white rhinoceros. However, individual traits like sex and age had no influence on the probability of being infected (Table S2).

### 4.4. Clinical Interventions

From October 2011 to March 2023, 26 clinical interventions related to stephanofilarial wounds were undertaken on rhinoceros in Kenya. Most (88.5%, *n* = 23/26) of the interventions were on white rhinoceros and the rest on black rhinoceros (11.5%, *n* = 3/26). This was significantly different from what would be expected based on the numerical abundance of each species (*X*_1_^2^ = 12.73, *P*=0.0004). All the black rhinos attended to were male. Among the white rhinos with known sex (21/23), 71.4% (15/21) were male and 28.6% (6/21) were female and the observed variation in clinical severity among the sexes was statistically significant from the expected 1 : 1 sex ratio (*X*_1_^2^ = 3.86, *P*=0.0495). About 81% (21/26) of all rhino treatment interventions came from MRS and about 83% (19/23) of all the white rhinoceros. Two rhinos were treated in SRS and a single individual each from OPC and LNP.

## 5. Discussion

The prevalence of stephanofilarial skin lesions among Kenyan rhinoceros populations varied from 0% in the lowland and moderate elevations consisting of NRS, IPZ, and TSE to >90% in the highland rhinoceros' sanctuaries including SRS, OPC, and LBL. The high prevalence of stephanofilarial skin lesions observed here are comparable to 84.3% prevalence in black rhinoceros population from the Zambezi Valley in Zimbabwe [9] and 100% prevalence in adult black rhinoceros from the Hluhluwe-iMfolozi Park (HiP), in South Africa [8]. A similar disease caused by *Stephanofilaria assamensis* in cattle was found to vary in prevalence from 10.2% in the Little Andaman island to 70.5% in the Middle Andaman islands, within the Andaman and Nicobar Island chain in India [7]. Similarly, the prevalence of *Stephanofilaria* was spatially variable in Queensland Australia; from less than 5% in south east Queensland to 95% on Cape York Peninsula while absent from most of the south west region [45].

Minimum monthly temperature was negatively related to the incidence of stephanofilarial skin lesions in Kenyan rhinoceros. These findings suggest that higher minimum temperature may limit the survival and proliferation of the nematodes and their fly vectors. Mihok et al. [14] studying flies associated with translocated rhinoceros infected with stephanofilarial wounds, found that *R. brucei* Malloch [13], a biting fly previously associated with skin lesions caused by *S. dinniki* [1] was not encountered in Ngulia (NRS) and Lugard's Falls (TSE). The two sanctuaries we found an absence of stephanofilarial wounds. Mihok et al. [14] also noted that stephanofilarial lesions present in animals translocated from Nairobi National Park healed a few weeks after the animals arrived at Ngulia (NRS). Similarly, they found that stephanofilarial skin lesions in animals translocated from Solio Ranch (currently SRS) healed quickly after confinement near Lugard Falls (in the TSE). In Solio and Nairobi, Mihok et al. [14] reported stephanofialarial wounds in translocated rhinoceros, and the presence of *R. dutoiti*, a species of fly previously found in Umfolozi and Hluhluwe Game Reserve in Kwazulu-Natal, South Africa and considered a potential vector for *S. dinniki*. In these same reserves, we reported a 96%–100% prevalence of stephanofilarial wounds in black rhinoceros. The Kenyan highlands and eastern South Africa are cooler than the arid locations of other rhinoceros reserves and such cooler temperatures appear ideal for the survival of *R. dutoiti*. A high prevalence of stephanofilarial wounds was observed in Solio and Nairobi, and notable absence in Tsavo East (TSE) and Ngulia (NRS). These results coincides with the presence of *R. dutoiti* in Solio and Nairobi and its absence in Tsavo East and Ngulia as revealed by Mihok et al. [14], and suggest that the distribution of *R. dutoiti* may be driving variation in the prevalence of stephanofilarial wounds across rhinoceros' sanctuaries in Kenya. Moreover, Mihok et al. [14] also observed that filarial wounds in rhinoceros translocated from Nairobi and Solio to Ngulia healed quickly perhaps as result of a lack of reinfection by the vector. However, further studies are required to determine the abundance of *R. dutoiti* and other potential vectors across rhinoceros' sanctuaries for conclusive results. The prevalence of cattle stephanofilariasis in Queensland Australia was highest in those areas where buffalo fly (vector) infestations were heaviest throughout the year, and lowest in those areas where fly numbers were low or seasonal [45]. In Oklahoma USA, positive relationships were found between the environmental temperatures, rainfall, horn fly (vector) populations, and average numbers of *Stephanofilaria stilesi* third stage larvae recovered from individual cattle, indicating that these factors affect the transmission of *S. stilesi* from vector to the host [18].

NDVI had a negative relationship with the incidence of stephanofilarial skin lesions in Kenyan rhinoceros. This result suggest that poor nutrition associated with low NDVI [46] could negatively impact host immunity, leading to increased susceptibility to infection [21, 47]. Support for this comes from a study in HiP, South Africa, where a temporal negative correlation was observed between severity of skin lesions and body condition score [8]. In a study on the incidence of lymphatic filaria transmitted by Anopheles mosquitos, in Burkina Faso, mean NDVI was also found to be negatively associated with the prevalence of lymphatic filariasis [27].

Spatial NDVI heterogeneity had positive relationships with the incidence of stephanofilarial skin lesions in rhinoceros. High heterogeneity in NDVI or standard deviation of NDVI, is a measure of diversity and variability in the structure of vegetation. Diverse and heterogeneous vegetation structure is known to be good habitat for black rhinoceros [48] and *R. dutoiti* as well. Studies in NNP revealed that the abundance of *R. dutoiti* was nearly threefold in woodland habitat compared to forest habitats [49]. In Burkina Faso, spatial heterogeneity or SD of NDVI was associated with a high prevalence in lymphatic filariasis, another disease transmitted by Anopheles mosquitos to humans [27].

Rhinoceros density was another factor positively related to the prevalence of stephanofilarial wounds in rhinoceros. *Rhinomusca*, a muscid vector of *Stephanofilaria* exclusively uses rhinoceros' dung as substrate to lay their eggs which hatch into larvae, and feed on the substrate until they pupate. A high density of rhinoceros corresponds to an elevated dung density, which directly impacts vector populations when other factors favorable for survival of adults, such as temperature, are suitable. For example, a study on factors driving lymphatic filariasis in Burkina Faso revealed that human population density and length of the rainy season were positively associated with the prevalence of Lymphatic filariasis in humans [27].

Black rhinoceros had a higher prevalence of stephanofilarial skin lesions than white rhinoceros. Except for observations from the MRS in Kenya and this study, no stephanofilarial wounds have been recorded for white rhinoceros in the wild [3]. The absence of reports may suggest that stephanofilarial infection is rare in white rhinoceros or that white rhinoceros have a higher resistance to *Stephanofilaria* than black rhinoceros. Studies of Stephanofilarial lesions in cattle have shown variation in susceptibility to infection among cattle breeds suggesting a genetic basis of resistance to infection. For example, a study in Australia found that *Bos indicus* cattle species had a lower prevalence to stephanofilarial lesions than *Bos taurus* probably due to their resistance which may have evolved during long evolutionary contact between *B. indicus* and the parasite and its vector [45]. The other reason for divergence in disease prevalence between black and white rhinoceros would be related to habitat differences between white and black rhinoceros. If these differences influence habitat suitability for the vector, it could be the major driver of the observed patterns. For example, black rhinoceros are known to favor habitats with thickets/shrubs and savannas with patches of short woody vegetation [50], while white rhinoceros have a strong preference for open grasslands [51]. Indeed, *Rhinomusca* a species suspected to be a vector of *Stephanofilaria* have been shown to have preference for woody vegetation habitats [49].

In contrast to the prevalence of stephanofilarial lesions, clinical interventions were more common in white rhinoceros than expected based on their occurrence among the two species. This observation suggests that although white rhinoceros seldom get infected, they may suffer debilitating clinical effects when infected. In agreement, Mutinda et al. [3] observed that filarial wounds were larger in white rhinoceros with a mean size of 23 ± 8 cm in diameter, while much smaller in black rhinoceros with a mean diameter of 15 ± 5 cm. These debilitating effects could also be related to the predilection of stephanofilarial wounds for the thigh in white rhinoceros (44%) compared to the black rhinoceros (6%). Such effects could affect mobility in infected white rhinoceros more than in black rhinoceros. Moreover, it should be noted that most white rhino infections leading to clinical interventions occurred in MRS suggesting perhaps some environmental stressors are present in MRS.

The prevalence of stephanofilarial skin lesions in black and white rhinoceros was not influenced by age-class, or sex. This finding deviates from results of previous studies in which wounds were larger and more prevalent in older animals compared to the young animals [7, 45, 52]. In cattle from the Nicobar Islands India, the overall prevalence of stephanofilarial wounds was higher in males (42.5%) than that in females (37.8%) and older animals had higher prevalence rate than young animals. Infection was not recorded in calves below 1 year of age [7, 45]. In Queensland Australia, the prevalence of lesions in cattle also varied with age from 70% in calves to 100% in adult animals [53]. The descripancy between our study and previous studies could be due to the narrow age range of animals that were sampled. Most animals sampled were from notching and these tended to be subadults and relatively young adults.

The predilection sites for stephanofilarial lesions were nonrandom in both black and white rhinoceros. In cattle, different species of *Stephanofialaria* have been shown to have consistent predilection sites on the body of the host [28]. In Malaya, the skin lesions caused by *Stephanofilaria kaeli* is invariably in the limbs of cattle [54] whereas in East Pakistan *Stephanofilaria assamensis*, is known to be associated with hump sore of cattle [55]. On the other hand, *Stephanofilaria stilesi* skin lesions are generally located along the ventral midline of the hosts particularly the skin of abdomen, udder, teat, scrotum, or flanks [17, 56]. In rhinoceros, most filarial wound sites were located around the chest with 55% of individuals having wounds at the lateral–posterior chest skin in black rhinoceros and on the hind legs or thighs in the white rhinoceros. These finding are consistent with results from a previous study in Kenya which found that white rhinoceros had filarial skin lesions on the rump, behind the shoulder and the axillary regions, while the black rhinoceros had filarial skin lesion on the ribs and behind the shoulder [3]. In Hluhluwe and Mkuzi Game Reserves most lesions were on the lateral anterior chest [29] similar to what was observed for the black rhinoceros during this study. In contrast, ventral neck lesions were observed in 100% of black rhinoceros in Hluhluwe Game Reserve [16] while in Zambezi valley, Zimbabwe 81.4% of lesions were on the ventral neck and only 2.3% were located at lateral posterior chest skin [9]. Several parasites in the genus *Stephanofilaria* cause skin lesions in livestock with predilections sites of lesions corresponding to the preferred feeding sites of parasitic insect vectors on the host [57, 58]. The differences in distribution of the lesions between black rhinoceros compared to white rhinoceros may suggest a different vector causing this disease or variation in *Stephanofilaria* strains infecting white and black rhinoceros.

## 6. Conclusion

Minimum monthly temperature, and NDVI spatial heterogeneity had statistically significant relationships with stephanofilarial wound prevalence. This was most likely due to these variables' influence on the distribution and abundance of filaria fly vector *R. dutoiti* suggested by prior studies. Moreover, the inverse relationship between monthly NDVI and stephanofilarial wound prevalence suggests that nutrition may be negatively impacting rhinoceros' immunity with positive outcomes for disease prevalence. Studies to establish factors influencing *Stephanofilaria* vector bionomics may illuminate the epidemiological patterns of stephanofilariosis. Similarly, studies examining the relationship between wound presence and severity in relation to body condition would directly shed light on the influence of nutrition on the epidemiology of stephanofilarial wounds. The severity of stephanofilarial wounds in MRS as reflected by clinical interventions requires further attention to understand factor causing the flaring up of infections in white rhinoceros.

## Figures and Tables

**Table 1:** Population estimates and numbers of black and white rhinoceros examined for Stephanofilarial skin lesion in rhinoceros sanctuaries in Kenya.

Population acronym	Population	Area of sanctuary (km^2^)	Population estimates		Number sampled	
			Black	White	Black	White
SRS	Solio Wildlife Conservancy^1^	76.9	75	500	17	16
OPC	Ol Pejeta Wildlife Conservancy^1^	93.0	166	44	24	21
LBL	Lewa/Borana Wildlife Conservancy^1^	376.36	141	123	23	21
NRS	Ngulia Rhino Sanctuary^2^	90.0	143	0	17	*0*
MRS	Meru Rhino Sanctuary	38.8	40	79	7	24
OLJ	Ol Jogi Wildlife Conservancy^1^	235	64	36	22	11
LNP	Lake Nakuru National Park	188.0	30	120	14	6
IPZ	Tsavo West National Park^2^	3000	37	0	31	0
TSE	Tsavo East National Park^2^	3300	24	0	24	0
NNP	Nairobi National Park	117.0	97	38	33	6
ALL	Kenya Metapopulation		817	940	228	110

^1^Population estimates from National Wildlife Census 2021. ^2^Population estimates for January 2023.

**Table 2:** Percentage of black (*n* = 121) and white (*n* = 18) rhinoceros in Kenya with stephanofilarial wounds at specific body locations (predilection sites).

Body part	Body part ID	Black rhinoceros	White rhinoceros	All rhinoceros species combined
Chest: lateral–anterior lower	S1	48.76	0	42.45
Chest: lateral–anterior upper	S2	49.59	0	43.17
Abdomen anterior	S3	32.23	22.22	30.94
Saddle	S4	0.83	0	0.72
Withers	S5	1.65	0	1.44
Lateral neck	S6	0	5.56	0.72
Ventral neck	S7	44.63	5.56	39.57
Chest: ventral–anterior	S8	2.48	5.56	2.88
Forelegs	S9	4.13	11.11	5.04
Shoulder	S10	16.53	16.67	16.55
Rump	S11	0	5.56	0.72
Tail base	S12	0	5.56	0.72
Chest: lateral posterior	S13	54.55	5.56	48.2
Flank: abdomen posterior	S14	1.65	0	1.44
Thigh	S15	5.79	44.44	10.07

**Table 3:** Prevalence of stephanofilarial skin lesions in black and white rhinoceros for each sanctuary in Kenya from 2021 to 2023.

Sanctuary	Numbers infected (*n*)	Numbers sampled (*N*)	Percent prevalence	95% Confidence interval for prevalence (lower CI–upper CI)
Eastern black rhinoceros (*Diceros bicornis michaeli*)				
IPZ	0	31	0	0–11.22
LNP	0	14	0	0–23.16
LBL	22	23	95.65	78.05–99.89
MRS	7	13	53.85	25.13–80.78
NNP	30	33	90.91	75.67–98.08
NRS	0	17	0	0–19.51
OLJ	7	20	35	15.39–59.22
OPC	33	36	91.67	77.53–98.25
SRS	17	17	100.00	17
TSE	0	24	0	0–14.25
Black rhinoceros	116	228	50.88	44.19–57.54
Southern white rhinoceros (*Ceratotherium simum simum*)				
IPZ		—	—	—
LNP	0	6	0	0–45.93
LBL	0	21	0	0–16.11
MRS	3	32	9.38	1.98–25.02
NNP	0	6	0	0–45.93
NRS	—	—	—	
OLJ	0	18	0	0–18.53
OPC	0	11	0	0–28.49
SRS	1	16	6.25	0.16–30.23
TSE	—	—	—	—
White rhinoceros	4	110	3.64	1–9.05

**Table 4:** Mean environmental parameters including total annual rainfall, monthly temperature, and monthly NDVI in 10 rhinoceros' sanctuaries in Kenya from June 2020 to May 2023.

Sanctuary	Minimum temperature ± SD	Maximum temperature ± SD	Mean temperature ± SD	Rainfall total ± SD	NDVI ± SD	Spatial heterogeneity in NDVI ± SD
IPZ	18.527 ± 1.475	29.077 ± 1.892	23.518 ± 1.487	430.818 ± 88.299	0.376 ± 0.120	0.079 ± 0.042
LNP	13.500 ± 0.930	23.891 ± 1.919	18.512 ± 1.350	954.585 ± 154.417	0.560 ± 0.129	0.073 ± 0.019
LBL	13.008 ± 0.715	22.405 ± 1.018	17.466 ± 0.690	946.318 ± 328.204	0.341 ± 0.115	0.058 ± 0.020
MRS	20.981 ± 0.834	31.044 ± 1.386	25.814 ± 1.045	383.995 ± 28.063	0.453 ± 0.156	0.080 ± 0.032
NNP	14.632 ± 1.130	24.245 ± 1.824	19.127 ± 1.306	409.866 ± 106.801	0.426 ± 0.119	0.090 ± 0.019
NRS	19.907 ± 1.457	30.055 ± 1.669	24.556 ± 1.394	346.472 ± 93.497	0.394 ± 0.136	0.054 ± 0.033
OLJ	13.228 ± 0.790	24.738 ± 1.214	18.902 ± 0.801	415.641 ± 186.035	0.354 ± 0.123	0.047 ± 0.015
OPC	12.054 ± 0.753	23.683 ± 1.506	17.669 ± 0.893	478.482 ± 196.664	0.463 ± 0.101	0.073 ± 0.015
SRS	12.220 ± 0.750	21.520 ± 1.466	16.551 ± 1.047	1325.614 ± 250.233	0.473 ± 0.105	0.089 ± 0.019
TSE	21.420 ± 1.340	31.524 ± 1.721	25.814 ± 1.406	253.929 ± 37.523	0.267 ± 0.076	0.057 ± 0.034

**Table 5:** Standardized estimates of model coefficients for best model based on AIC predicting the occurrence of filarial lesions in eastern black and southern white rhinoceros in Kenya.

Parameter	Estimate	Std. error	*z* Value	*P* value	Odds ratio	Odds ratio 95% confidence interval
Intercept	0.349	0.246	1.418	0.1563	1.418	0.875–2.309
Minimum temperature (celsius)	−1.219	0.232	−5.250	<0.0001	0.296	0.183–0.457
Mean monthly NDVI	−1.216	0.334	−3.642	0.0003	0.296	0.143–0.539
Spatial NDVI heterogeneity	0.786	0.309	2.544	0.0110	2.194	1.211–4.101
Rhinoceros density (number/km^2^)	1.650	0.389	4.242	<0.0001	5.205	2.503–11.513
White cf. black rhinoceros	−9.564	1.700	−5.627	<0.0001	0.000	0.000–0.002

## Data Availability

All the relevant data have been included in the main manuscript or as supplementary materials.

## Supplementary Materials

Supplementary Materials Table S1: mean of climatic variables from June 2016 to May 2023 for all the major rhinoceros' sanctuaries in Kenya. Table S2: bivariate logistic regression models showing covariates predicting prevalence of stephanofilarial skin lesions.
