# Supplementary material for: Identifying Risk Factors for Stephanofilaria-Caused Ulcerative Dermal Lesions, in Black and White Rhinoceros' Meta-Population in Kenya
Source: Transbound Emerg Dis. 2024 Jan 10;2024:2655970. doi: 10.1155/2024/2655970 (PMC12016875; doi:10.1155/2024/2655970)
Supplement: Supplementary Materials — Table S1: mean of climatic variables from June 2016 to May 2023 for all the major rhinoceros' sanctuaries in Kenya. Table S2: bivariate logistic regression models showing covariates predicting prevalence of stephanofilarial skin lesions. [file 2655970.f1.docx]

**Identifying risk factors for *Stephanofilaria*-caused ulcerative dermal lesions in black and white rhinoceros’ meta-population in Kenya**

**Identifying risk factors for *Stephanofilaria*-caused ulcerative dermal lesions in black and white rhinoceros’ meta-population in Kenya.**

Edward King’ori¹, Grace Waiguchu², Mukami Ruoro¹, Kenneth Muriithi^3^, Cecilia Mumbi^4^, Martin Omondi^3^, Duncan Aminga¹, Shaleen Angwenyi⁵, Domnic Mijele^1^, Patrick I. Chiyo^6^*****

**Author Affiliations**

¹ Veterinary Department, Kenya Wildlife Service, P.O. Box 40241 – 00100 Nairobi Kenya

² Research Division, Wildlife Research and Training Institute, PO Box 842-20117 Naivasha, Kenya

³ Wildlife Security Directorate, Kenya Wildlife Service, P.O. Box 40241 – 00100 Nairobi Kenya

⁴ Natural State Research Centre, Private bag Isiolo, Kenya

^5^ Global Health Program, Smithsonian Conservation Biology Institute, Washington, DC, USA

^6*^ Wildlife Genetics and Forensics Laboratory, Kenya Wildlife Service, P.O. Box 40241 – 00100 Nairobi Kenya

***Corresponding author and Address**

**Supplementary Tables**

Two supplementary data tables are provided in this supplementary document. Table S1 provides the average values of climatic variables in the focal rhino sanctuaries over an eight-year period starting from June 2016 and ending in May 2023. Table S2 consists of parameters of several bivariate logistic regression models. The dependent variable is the presence or absence of infection with stephanofilarial wounds, and the several independent variables shown including among others, monthly rainfall, monthly Normalized Difference Vegetation Index (NDVI) values, Monthly Standard Deviation in spatial NDVI values, rhinoceros’ species (whites vs black), Sex (male vs

female), Age class (Juvenile/Infant, Sub-adult, and Adult) and Monthly temperature (average daily minimum, maximum and mean)

**Table S1:** mean of climatic variables from June 2016 to May 2023 for all the major rhinoceros’

sanctuaries in Kenya

| **Sanctuary** | **Mean temperature** | **Minimum temperature** | **Maximum temperature** | **NDVI** | **Spatial heterogeneity in NDVI** | **Total Annual rainfall** |
| --- | --- | --- | --- | --- | --- | --- |
| **IPZ** | 23.4 ± 1.4 | 18.6 ± 1.445 | 28.8 ± 1.9 | 0.39 ± 0.13 | 0.085 ± 0.04 | 615.6 ± 259.8 |
| **LNP** | 18.6 ± 1.4 | 13.6 ± 0.914 | 24.0 ± 2.0 | 0.57 ± 0.14 | 0.073 ± 0.02 | 1088.4 ± 381.4 |
| **LBL** | 17.4 ± 0.8 | 13.1 ± 0.817 | 22.2 ± 1.1 | 0.37 ± 0.13 | 0.057 ± 0.02 | 1252.7 ± 554.9 |
| **MRS** | 25.7 ± 1.0 | 21.0 ± 0.77 | 30.9 ± 1.4 | 0.46 ± 0.16 | 0.077 ± 0.03 | 613.8 ± 282.3 |
| **NNP** | 19.1 ± 1.3 | 14.7 ± 1.125 | 24.2 ± 1.8 | 0.43 ± 0.12 | 0.088 ± 0.02 | 620.5 ± 380.1 |
| **NRS** | 24.5 ± 1.3 | 20.1 ± 1.429 | 29.8 ± 1.6 | 0.42 ± 0.16 | 0.051 ± 0.03 | 568.2 ± 298.1 |
| **OLJ** | 18.8 ± 0.9 | 13.3 ± 0.742 | 24.5 ± 1.4 | 0.38 ± 0.12 | 0.050 ± 0.02 | 674.6 ± 409.2 |
| **OPC** | 17.5 ± 1.0 | 12.1 ± 0.772 | 23.3 ± 1.6 | 0.48 ± 0.11 | 0.077 ± 0.02 | 860.0 ± 483.6 |
| **SWS** | 16.3 ± 1.1 | 12.2 ± 0.824 | 21.1 ± 1.5 | 0.49 ± 0.13 | 0.085 ± 0.02 | 1780.3 ± 686.6 |
| **TSE** | 25. 7 ± 1.3 | 21.4 ± 1.31 | 31.2 ± 1.7 | 0.29 ± 0.10 | 0.060 ± 0.04 | 419.1 ± 203.6 |

**Table S2:** Bivariate logistic regression models showing covariates predicting prevalence of

stephanofilarial skin lesions.

| **Variable** | **Coefficient Estimate** | **Std. Error** | **z value** | **Pr(>\|z\|)** | **AIC** |
| --- | --- | --- | --- | --- | --- |
| **Intercept** | -0.128 | 0.127 | -1.011 | 0.31200 | 384.96 |
| **Species (White cf. Black rhinoceros** | -3.281 | 0.524 | -6.265 | < 0.0001 |  |
| **Intercept** | -1.023 | 0.144 | -7.127 | < 0.0001 | 392.75 |
| **Minimum Temperature** | -1.225 | 0.162 | -7.576 | < 0.0001 |  |
| **Intercept** | -0.925 | 0.131 | -7.084 | < 0.0001 | 409.07 |
| **Mean Temperature** | -1.030 | 0.144 | -7.176 | < 0.0001 |  |
| **Intercept** | -0.841 | 0.121 | -6.934 | < 0.0001 | 430.62 |
| **Maximum Temperature** | -0.786 | 0.125 | -6.288 | < 0.0001 |  |
| **Intercept** | -0.761 | 0.113 | -6.743 | < 0.0001 | 462.98 |
| **Rhinoceros Density** | 0.382 | 0.108 | 3.529 | 0.00042 |  |
| **Intercept** | -0.791 | 0.116 | -6.810 | < 0.0001 | 460.97 |
| **Monthly mean NDVI** | -0.495 | 0.144 | -3.429 | 0.0006 |  |
| **Intercept** | -0.770 | 0.114 | -6.770 | < 0.0001 | 467.4 |
| **Monthly rainfall** | -0.381 | 0.149 | -2.547 | 0.01090 |  |
| **Intercept** | -0.720 | 0.163 | -4.416 | < 0.0001 | 475.61 |
| **Sex: Male cf. Female** | -0.041 | 0.222 | -0.183 | 0.85500 |  |
| **Intercept** | -0.718 | 0.193 | -3.722 | 0.0002 | 476.7 |
| **Age Class: Calf cf. Adult** | -0.234 | 0.317 | -0.740 | 0.45943 |  |
| **Age Class: Sub-Adult cf. Adult** | 0.050 | 0.251 | 0.201 | 0.84052 |  |
| **Intercept** | 0.323 | 0.024 | 13.674 | < 0.0001 | 480.82 |
| **Spatial heterogeneity in NDVI** | 0.104 | 0.024 | 4.416 | < 0.0001 |  |
